# Supplementary material for: Glycovariant-based lateral flow immunoassay to detect ovarian cancer–associated serum CA125
Source: Commun Biol. 2020 Aug 21;3:460. doi: 10.1038/s42003-020-01191-x (PMC7442799; doi:10.1038/s42003-020-01191-x)
Supplement: Supplementary file 1 — Supplementary Information [file 42003_2020_1191_MOESM1_ESM.pdf]

Supplementary figures

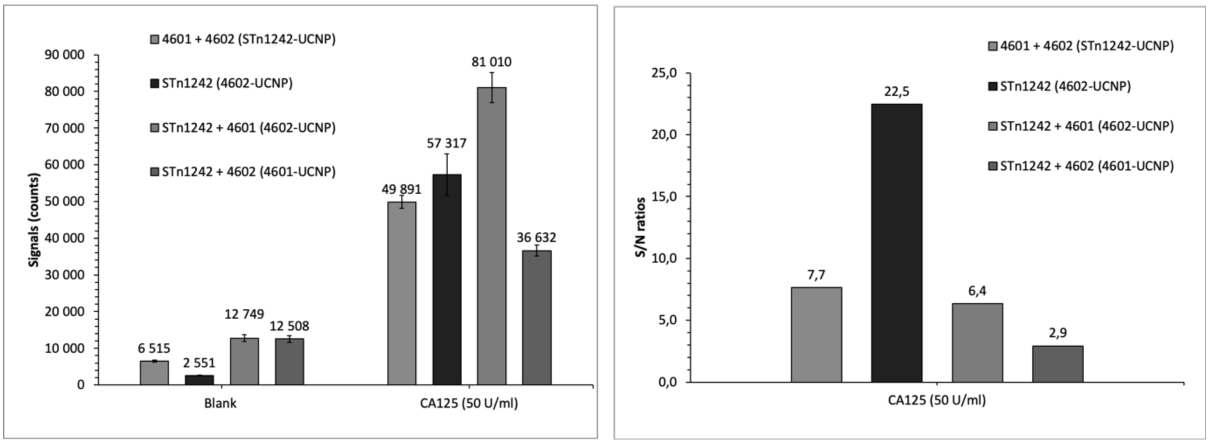

Supplementary Fig. 1 | Signal levels produced in the testing of different antibody combinations. The model analyte was spiked at a concentration of 50 U/mL in healthy serum samples. For each combination, the capture antibody or combination of antibodies are mentioned first, while the tracer bioconjugate is shown in parentheses. Error bars represent standard deviation of three replicates of each test.

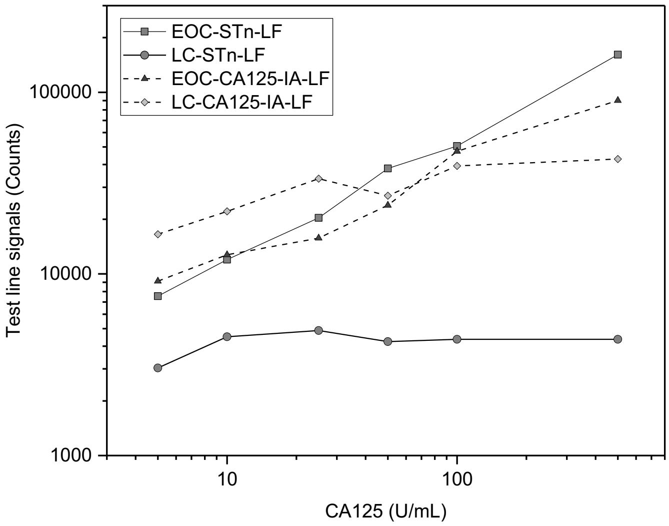

Supplementary Fig. 2 | Comparison between the conventional approach and the glycovariant-based approach using a lateral flow platform and UCNPs as label. In CA125-STn-LF, EOC-CA125 depicted similar specific signals with that observed for CA125^{IA}-LF. However, LC-CA125 showed almost negligible signal levels with CA125-STn-LF assay, while CA125-IA-LF showed interfering signals and an overlap between liver cirrhosis-CA125 (LC-CA125) and ovarian cancer spiked samples.

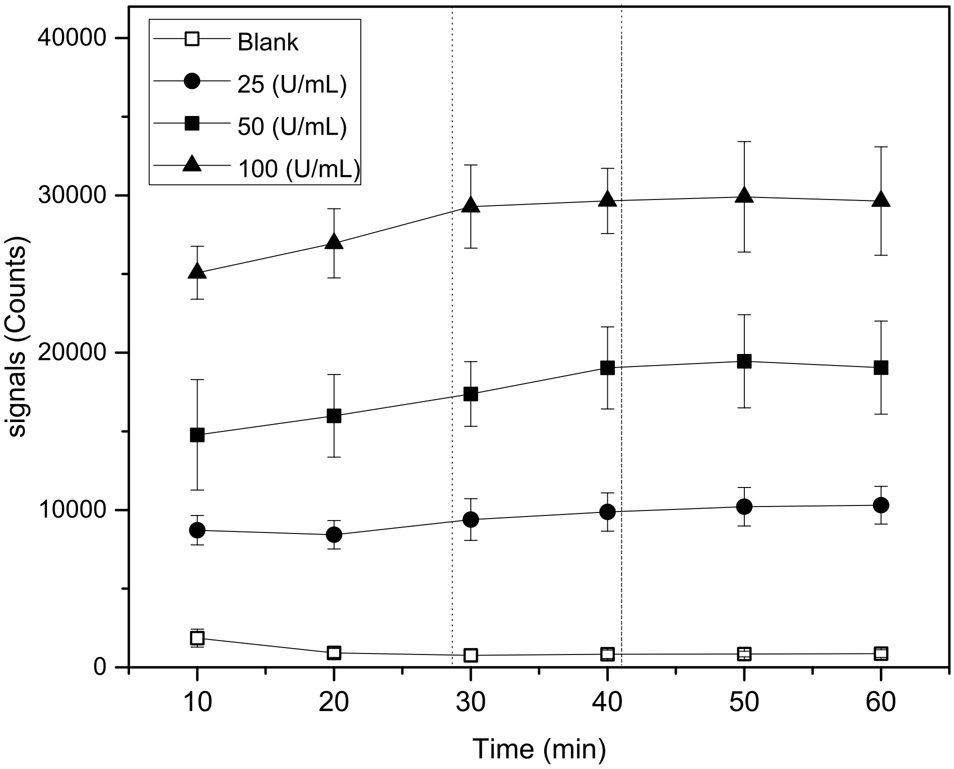

Supplementary Fig. 3 | Signal response to blank calibrator or spiked calibrators (25, 50, and 100 U/mL) when the assay is run. The upconversion luminescence (UCL) was measured at 540 nm upon 976 nm excitation. The error bars represent the standard deviations of six replicates of each calibrator at different time intervals.

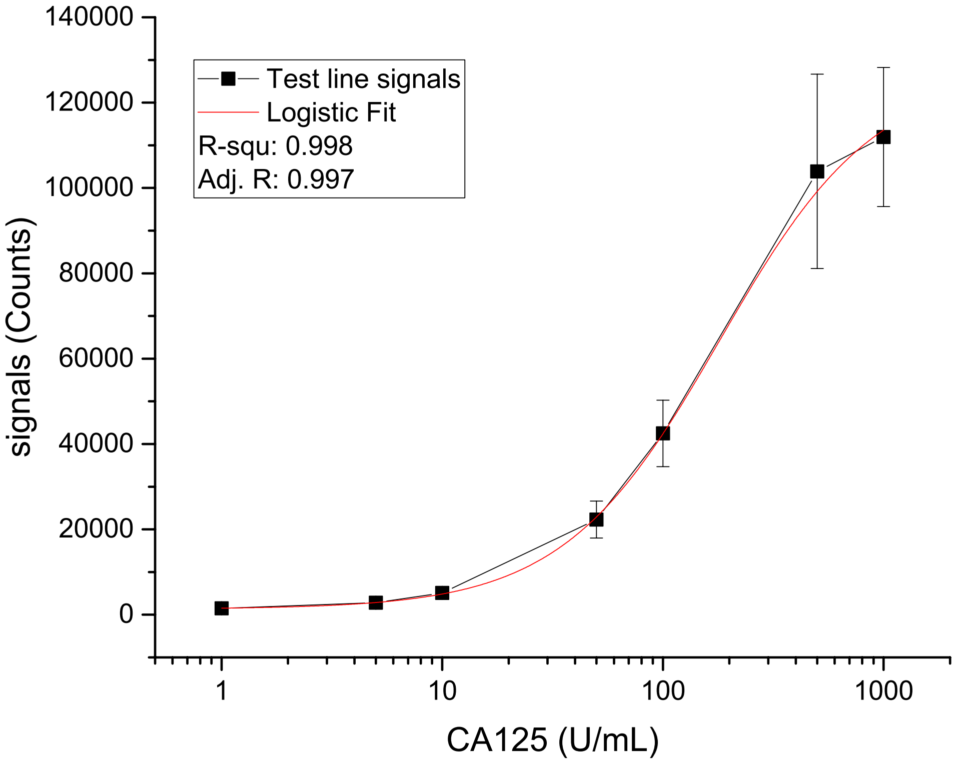

Supplementary Fig. 4 | Calibration curve of the developed CA125-STn-LFIA (solid line). The dynamic range of the test was shown to be at 1–1000 U/mL. Error bars represent standard deviation calculated using the specified number of the calibrator using the specified number of replicates.

Supplementary Tab. 1 | Patient characteristics

	N	Age, y<br>mean	CA125-IA (U/ml)<br>mean ± SD	CA125-LFIA (U/ml)<br>mean ± SD
Healthy	40	27.2	10.8 ± 3.3	0.4 ± 0.96 (<LoD)
Endometriosis	31	36	90.6 ± 43.1	1.1 ± 2.96 (<LoD)
Ovarian cancer*	41	63.8	91.9 ± 50.9	19.4 ± 19.1
Stage (FIGO 2014)				
III	17			
IV	14			

* High-grade serous ovarian cancer (100%)
