# Supplementary material for: Glycovariant-based lateral flow immunoassay to detect ovarian cancer–associated serum CA125
Source: Commun Biol. 2020 Aug 21;3:460. doi: 10.1038/s42003-020-01191-x (PMC7442799; doi:10.1038/s42003-020-01191-x)
Supplement: Supplementary file 3 — Description of Additional Supplementary Files [file 42003_2020_1191_MOESM3_ESM.pdf]

Description of Additional Supplementary Files

File Name: **Supplementary Data 1**

Description: The excel sheet contains the quantitative concentrations of CA125 in the tested samples, in (U/mL), which were measured using the conventional CA125 reference ELISA in one column. The healthy and endometriosis samples were coded as (0) to plot ROC and box plot curves, while ovarian cancer samples were coded (1). The other column contains the upconversion absorbance measured using the developed lateral flow test. plotting the measured signals would give the reader more profound perspective about the findings.
