# Supplementary material for: Glycovariant-based lateral flow immunoassay to detect ovarian cancer–associated serum CA125
Source: Commun Biol. 2020 Aug 21;3:460. doi: 10.1038/s42003-020-01191-x (PMC7442799; doi:10.1038/s42003-020-01191-x)
Supplement: Supplementary file 4 — Reporting Summary [file 42003_2020_1191_MOESM4_ESM.pdf]

nature research

Corresponding author(s): Kim Pettersson

Last updated by author(s): Jul 16, 2020

Reporting Summary

Nature Research wishes to improve the reproducibility of the work that we publish. This form provides structure for consistency and transparency in reporting. For further information on Nature Research policies, see Authors & Referees and the Editorial Policy Checklist.

Statistics

For all statistical analyses, confirm that the following items are present in the figure legend, table legend, main text, or Methods section.

n/a	Confirmed	
☐	☒	The exact sample size (*n*) for each experimental group/condition, given as a discrete number and unit of measurement
☐	☒	A statement on whether measurements were taken from distinct samples or whether the same sample was measured repeatedly
☐	☒	The statistical test(s) used AND whether they are one- or two-sided<br>*Only common tests should be described solely by name; describe more complex techniques in the Methods section.*
☐	☒	A description of all covariates tested
☐	☒	A description of any assumptions or corrections, such as tests of normality and adjustment for multiple comparisons
☐	☒	A full description of the statistical parameters including central tendency (e.g. means) or other basic estimates (e.g. regression coefficient) AND variation (e.g. standard deviation) or associated estimates of uncertainty (e.g. confidence intervals)
☐	☒	For null hypothesis testing, the test statistic (e.g. *F*, *t*, *r*) with confidence intervals, effect sizes, degrees of freedom and *P* value noted<br>*Give P values as exact values whenever suitable.*
☒	☐	For Bayesian analysis, information on the choice of priors and Markov chain Monte Carlo settings
☒	☐	For hierarchical and complex designs, identification of the appropriate level for tests and full reporting of outcomes
☒	☐	Estimates of effect sizes (e.g. Cohen's *d*, Pearson's *r*), indicating how they were calculated

Our web collection on statistics for biologists contains articles on many of the points above.

Software and code

Policy information about availability of computer code

Data collection	*Provide a description of all commercial, open source and custom code used to collect the data in this study, specifying the version used OR state that no software was used.*
Data analysis	*Provide a description of all commercial, open source and custom code used to analyse the data in this study, specifying the version used OR state that no software was used.*

For manuscripts utilizing custom algorithms or software that are central to the research but not yet described in published literature, software must be made available to editors/reviewers. We strongly encourage code deposition in a community repository (e.g. GitHub). See the Nature Research guidelines for submitting code & software for further information.

Data

Policy information about availability of data

All manuscripts must include a data availability statement. This statement should provide the following information, where applicable:

- Accession codes, unique identifiers, or web links for publicly available datasets
- A list of figures that have associated raw data
- A description of any restrictions on data availability

Data is available upon request, we can provide a coded patient number that refers to every sample with no further clinical details about the patients as agreed with the other parties.

Field-specific reporting

Please select the one below that is the best fit for your research. If you are not sure, read the appropriate sections before making your selection.

☒ Life sciences ☐ Behavioural & social sciences ☐ Ecological, evolutionary & environmental sciences

For a reference copy of the document with all sections, see nature.com/documents/nr-reporting-summary-flat.pdf

Life sciences study design

All studies must disclose on these points even when the disclosure is negative.

Sample size	No sample size calculation was performed in this study. however, samples were analyzed using the reference kit and out of these samples, endometriosis with concentrations below the cutoff, 35 U/mL, were excluded from the comparison to challenge both tests with these samples.
Data exclusions	EOC and endometriosis samples with conventional CA125 levels <35 or >200 U/ml were excluded due to focus of the study to the clinically challenging sample group with marginally elevated CA125 values.
Replication	The samples were measured as triplicates unless otherwise stated.
Randomization	Randomization was not applicable because only direct measures from the equipment were used for the statistical analysis. Any subjective assessment were not performed.
Blinding	Blinding was not applicable because only direct measures from the equipment were used for the statistical analysis. Any subjective assessment were not performed.

Reporting for specific materials, systems and methods

We require information from authors about some types of materials, experimental systems and methods used in many studies. Here, indicate whether each material, system or method listed is relevant to your study. If you are not sure if a list item applies to your research, read the appropriate section before selecting a response.

Materials & experimental systems

n/a	Involved in the study	
☐	☒	Antibodies
☒	☐	Eukaryotic cell lines
☒	☐	Palaeontology
☒	☐	Animals and other organisms
☐	☒	Human research participants
☒	☐	Clinical data

Methods

n/a	Involved in the study	
☒	☐	ChIP-seq
☒	☐	Flow cytometry
☒	☐	MRI-based neuroimaging

Antibodies

Antibodies used	Anti-cancer antigen 125: 4602-mAb (Cat. No. 100598) from Oy Medix Biochemica (Finland), Anti-cancer antigen 125: 4601-mAb (Cat. No. 628) from Oy Medix Biochemica (Finland). Anti-sialylated Tn antigen STn1242-mAb (CAT. No. 119-01R) from Fujirebio Diagnostics AB, Sweden). Polyclonal Rabbit Anti-Mouse Immunoglobulins: RAM for the control line of the developed lateral flow strips (code No. Z 0259), Dako Denmark AS (Glostrup, Denmark).
Validation	*Describe the validation of each primary antibody for the species and application, noting any validation statements on the manufacturer's website, relevant citations, antibody profiles in online databases, or data provided in the manuscript.*

Human research participants

Policy information about studies involving human research participants

Population characteristics	Participants are Caucasian women with epithelial ovarian cancer (EOC) or endometriosis. In addition, healthy volunteers were included in the study. EOC patients were treated according to the guidelines i.e. with debulking surgery and platinum-based chemotherapy.
Recruitment	Participants were prospectively recruited at the Department of Obstetrics and Gynecology at the Turku University Hospital in Finland from 2010 to 2013. Patients with abnormal pelvic processes and/or elevated serum CA125 were included. Further, only patients with histologically confirmed disease were included in the study. The study population consisted of xx patients with marginally elevated CA125 (35-200 U/ml), of which xx patients were diagnosed with EOC and xx patients with endometriosis. In

addition, healthy volunteers (n = xx) were included in the current study. Tissue samples were obtained during surgery and the histopathological diagnosis and disease stage were affirmed by a pathologist specialized in gynecological pathology. All patients participating in the study gave written informed consent.

Ethics oversight	The Ethics Committees of the Hospital District of Southwest Finland and the University of Turku, Turku, Finland approved the use of clinical materials applied.

Note that full information on the approval of the study protocol must also be provided in the manuscript.
